# Exogenous cathepsin G upregulates cell surface MHC class I molecules on immune and glioblastoma cells

**DOI:** 10.18632/oncotarget.12980

**Published:** 2016-10-28

**Authors:** Madleen Giese, Nadine Turiello, Nicole Molenda, David Palesch, Annika Meid, Roman Schroeder, Paola Basilico, Charaf Benarafa, Marc-Eric Halatsch, Michal Zimecki, Mike-Andrew Westhoff, Christian Rainer Wirtz, Timo Burster

**Affiliations:** ^1^ Department of Neurosurgery, Ulm University Medical Center, Ulm, Germany; ^2^ Institute of Molecular Virology, Ulm University Medical Center, Ulm, Germany; ^3^ Theodor Kocher Institute, University of Bern, Bern, Switzerland; ^4^ Graduate School for Cellular and Biomedical Sciences, University of Bern, Bern, Switzerland; ^5^ Ludwik Hirszfeld Institute of Immunology and Experimental Therapy, Polish Academy of Sciences, Wroclaw, Poland; ^6^ Department Pediatrics and Adolescent Medicine, Ulm University Medical Center, Ulm, Germany

**Keywords:** cathepsin G, MHC class I, glioblastoma stem cells, lactoferrin, CatG deficient mice, Immunology and Microbiology Section, Immune response, Immunity

## Abstract

Major histocompatibility complex (MHC) class I molecules present antigenic peptides to cytotoxic T cells. During an adaptive immune response, MHC molecules are regulated by several mechanisms including lipopolysaccharide (LPS) and interferon gamma (IFN-g). However, it is unclear whether the serine protease cathepsin G (CatG), which is generally secreted by neutrophils at the site of inflammation, might regulate MHC I molecules. We identified CatG, and to a higher extend CatG and lactoferrin (LF), as an exogenous regulator of cell surface MHC I expression of immune cells and glioblastoma stem cells. In addition, levels of MHC I molecules are reduced on dendritic cells from CatG deficient mice compared to their wild type counterparts. Furthermore, cell surface CatG on immune cells, including T cells, B cells, and NK cells triggers MHC I on THP-1 monocytes suggesting a novel mechanism for CatG to facilitate intercellular communication between infiltrating cells and the respective target cell. Subsequently, our findings highlight the pivotal role of CatG as a checkpoint protease which might force target cells to display their intracellular MHC I:antigen repertoire.

## INTRODUCTION

Foreign antigens are presented by major histocompatibility complex (MHC) molecules to activate T cells. The proteasome degrades cytosolic antigens and the resulting antigenic peptides are directed into the endoplasmatic reticulum (ER), further processed by exopeptidases, loaded to MHC I molecules, and the MHC I-peptide complex is presented on the cell surface to cytotoxic T lymphocytes (CTLs) [1]. In professional antigen presenting cells (APCs) a cross talk between endo- and exogenous antigens and MHC I-mediated antigen presentation exists. For instance, endocytosed cell surface MHC I molecules can be recycled, some ER resident MHC I molecules, which traffic to the trans-Golgi network, can be forced to the endocytic compartment, or MHC I molecules can be partially sorted to the phagosome. In these different compartments, MHC I molecules are possibly loaded with a new set of exogenous/endocytic-derived antigenic peptides for CTLs inspection [2–5].

Tumor cells downregulate expression of MHC I molecules to escape detection by CTLs, a mechanism known as immune evasion [6]. Glioblastoma cells, representing the most aggressive tumor in the brain, avoid the presentation of tumor-associated antigens and subsequent activation of CTLs by partly downregulating MHC I [7]. In order to evade detection by natural killer (NK) cells, glioblastoma cells maintain a limited set of MHC I at the cell surface [8]. The question remains how MHC I molecules could be restored on the cell surface to present tumorigenic antigens for recognition by CTLs.

During inflammation, polymorphonuclear neutrophils secrete different serine proteases, such as cathepsin G (CatG), neutrophil elastase (NE), and proteinase 3 (PR3), to promote an immune response [9]. Thereby secreted CatG can bind to the cell surface of T cells, B cells, and NK cells as well as activated pro-inflammatory monocytes [10, 11] and perform several physiological roles, including lymphocyte stimulation [12], generation of angiotensin II [13], or inactivation of the chemokine stromal cell-derived factor 1 (SDF-1) [11]. Furthermore, CatG bound on B cells can be internalized to the endocytic compartment to broaden their protease repertoire which is important for antigen processing [14]. However, it is not known whether exogenous CatG may regulate antigen presentation via MHC I on the target cell.

Lactoferrin (LF) is a multifunctional protein, contained in excretory fluids of mammals and secondary granules of neutrophils, playing a key role in innate and adaptive immunity [15]. Interestingly, LF was found to inhibit growth of glioblastoma cells, which form one of the most aggressive tumors, by a direct inhibition of the cell cycle [16]. However, until now, no data are available regarding LF as a possible enhancer of MHC class I cell surface expression. We found that LF enhances CatG activity and lowers its substrate specificity [17]. Therefore, investigation on potential additive or synergistic action by LF and CatG in regulation of MHC I is of interest.

Recently, we found that endogenous CatG, resident in the endocytic compartment, proteolytically degrades MHC I molecules [18]. Here, we demonstrate that exogenous CatG upregulates MHC I on the cell surface of different cell types, most likely, via deactivation of the protease-activated receptor 1 (PAR1). CatG-mediated upregulation of MHC I is further enhanced by LF in peripheral blood mononuclear cells (PBMCs) and a B cell line but not in glioblastoma stem cells. PBMCs, consisting, among others, of T cells, B cells, and NK cells and exhibit CatG on the cell surface [10] which, induced cell surface expression of MHC I molecules in THP-1. Moreover, dendritic cells (DCs) from CatG-deficient mice harbor less MHC I compared to their control counterparts. These data suggest that exogenous CatG regulates cell surface MHC I expression to support monitoring the intracellular status of target cells.

## RESULTS

### The proteolytic activity of exogenous CatG elevates cell surface expression of MHC I

**Figure 1:**
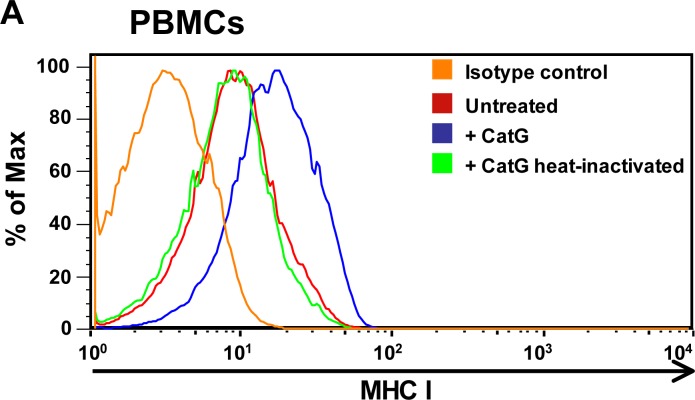

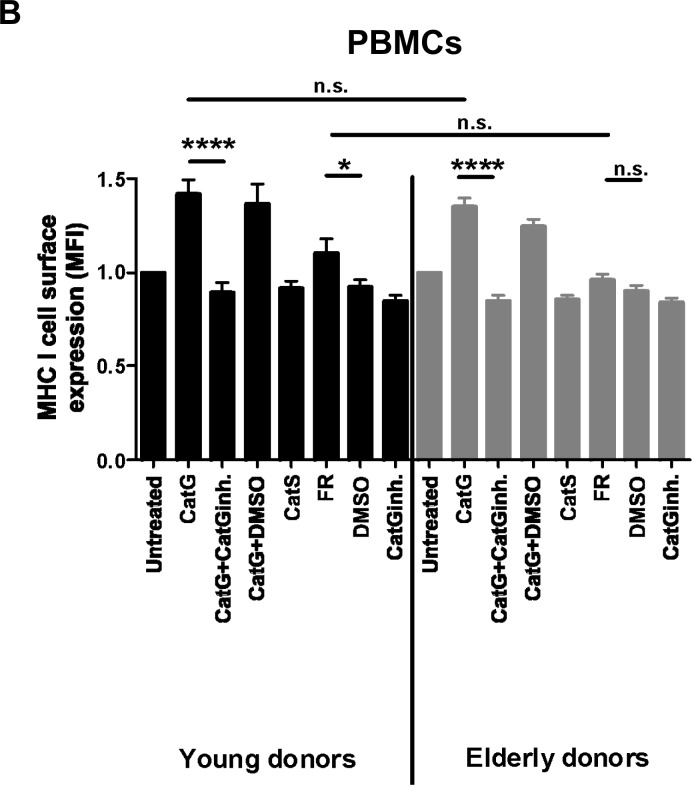
Analysis of cell surface expression of MHC I molecules **A.** Human peripheral blood mononuclear cells (PBMCs) were incubated for 6 h with either CatG or heat inactivated CatG and the MHC I cell surface expression was analyzed by using flow cytometry. Data of one representative experiment out of five is shown. **B.** PBMCs were obtained from young and elderly donors and were incubated with CatG, CatS, FR, or the respective inhibitor. Cells were gated for lymphocytes. Data were normalized and considered significant at *p* < 0.05 (*), *p* < 0.0001 (****), and not significant at *p* > 0.05 (n.s.) by using the unpaired two-tailed Student's *t* test. Error bars indicate the standard error of the median (SEM). A total of ten experiments (*n* = 10 young donors; *n* = 10 elderly donors) were performed. inh. = inhibitor.

CatG is secreted by neutophils at the site of inflammation to promote a rapid immune response. As a result, CatG can bind to the cell surface of several types of immune cells [9–11]. This raises the question whether exogenous CatG might induce the expression of accessory molecules on target cells. To this end, human peripheral blood mononuclear cells (PBMCs) were incubated with purified human CatG and cell surface molecules were analyzed by flow cytometry. We found that MHC I was upregulated by active CatG, but not by heat inactivated CatG (Figure 1A), which was added to the assay to exclude the presence of a non-protease contamination of the CatG preparation. In another control experiment, several different inhibitors were used to verify the specificity of CatG. The inhibitor panel included the reversible CatG inhibitor, CatG inhibitor I, and the irreversible CatG inhibitor (SucVPF) which reduced CatG-induced MHC I expression, compared to the cysteine protease inhibitor, E64 or the aspartic protease inhibitor, PepA which did not affect CatG activity (Supp. data S1). Thus, the proteolytic activity of CatG provokes an increase of MHC I on the cell surface of PBMCs.

Protease-activated receptors (PARs) belong to the family of G-protein-coupled receptors. CatG, for instance, cleaves PAR1-4 which leads to the activation of the receptor and followed by a wide range of cellular functions. However, CatG can also inactivate (disarm) PAR depending on the cleavage motif thereby switching on different pathways or disable signaling [19, 20]. To investigate the potential mechanism of CatG-induced MHC I expression, human acute monocytic leukemia cell line (THP-1), which only expresses PAR1 and PAR4 [21], was incubated with the PAR1 antagonist (FR171113, FR) [22] or the PAR4 antagonist (tcY-NH_2_) [23] in the presence or absence of CatG. FR increased cell surface MHC I expression and was even further enhanced by adding CatG, compared to the PAR4 antagonist tTcY-NH_2_ which had no effect on cell surface MHC I (Supp. Data S2). In the next set of experiments, PBMCs from young or elderly donors, which do express PAR1 (Supp. Data S3), were used to determine possible differences in MHC I regulation depending on age. PBMC were incubated with CatG or the respective controls as described before. While CatG induced an increase of MHC I on the cell surface of PBMCs no significant differences between the two groups were detected (Figure 1B). Additionally, incubation of PBMCs with the PAR1 antagonist FR resulted in a similar upregulation of MHC I in young donors, whereas recombinant CatS or the vehicle control DMSO had no effect. Taken together, these results show that CatG-mediated abundance of MHC I are most likely due to the deactivation of PAR1.

### Lactoferrin-mediated enhancement of CatG activity elevates MHC I

**Figure 2:**
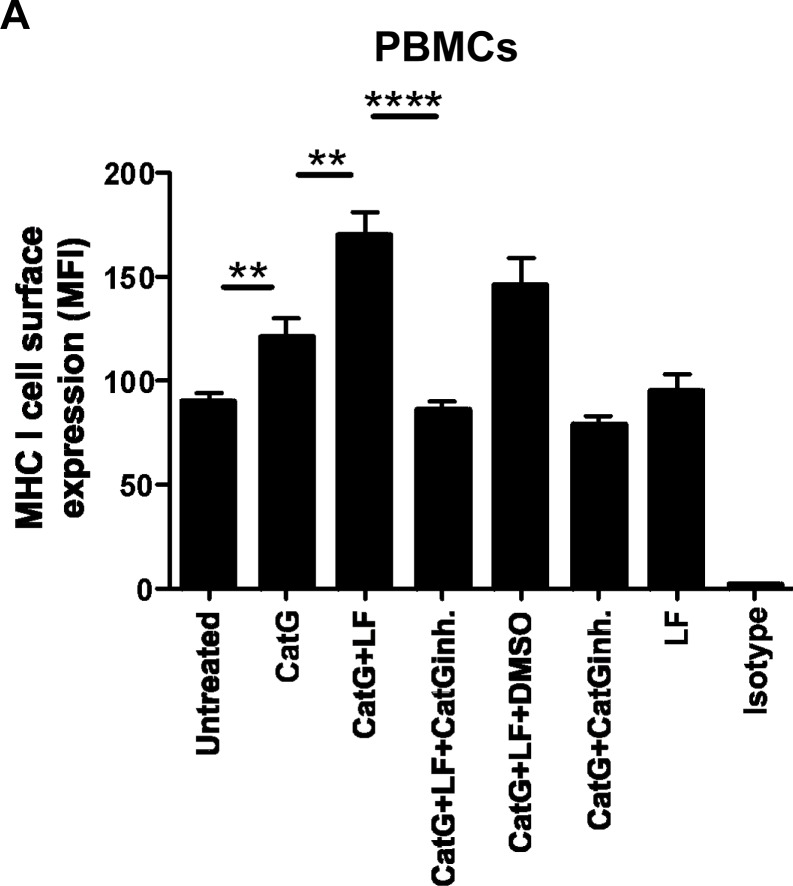

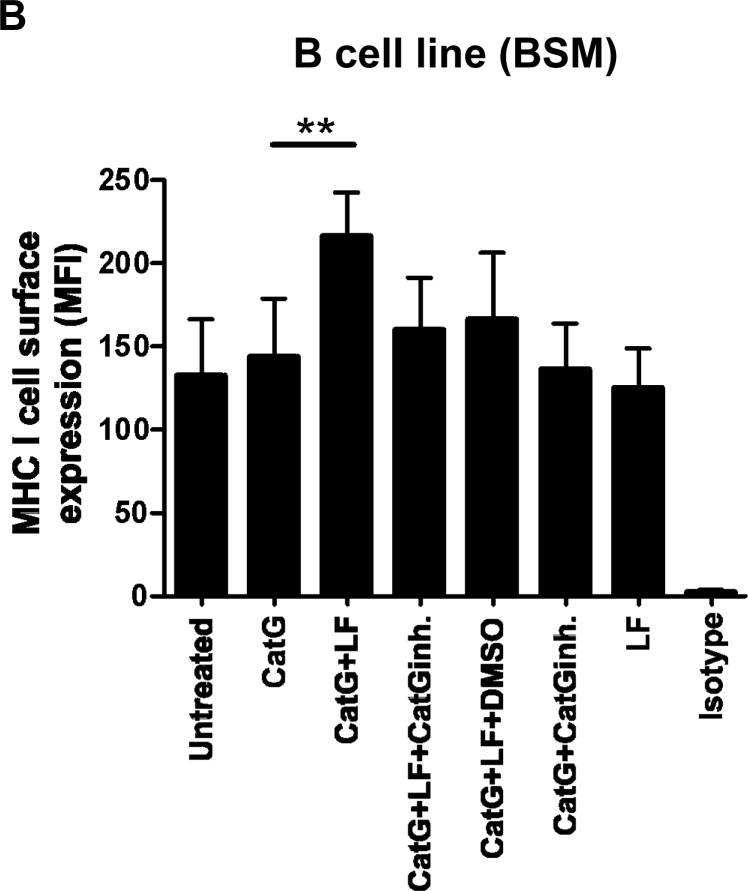
Detection of CatG-mediated enhancement of cell surface MHC I under the control of lactoferrin (LF) **A.** PBMCs or **B**. the B cell line (BSM) were incubated with CatG, CatG with LF, CatG with LF and CatG inhibitor (CatGinh.), CatG with LF and DMSO, or CatG with CatGinh. for 6h at 37°C. Cell surface expression of MHC I was determined by flow cytometry. Seven independent experiments were performed for PBMCs (*n* = 7) and six for BSM (*n* = 6).

Recently, we found that physiological concentration of lactoferrin (LF) enhanced the activity and broadens the substrate selectivity of CatG [17]. Having this in mind, we sought to determine whether the expression of MHC I can be further elevated by using CatG in combination with LF. CatG initiated an upregulation of MHC I at the cell surface of PBMCs as expected (Figure 2A). Strikingly, levels of MHC I were further increased by the combined action of CatG and LF. This is in contrast to the B cell line BSM where CatG did not significantly alter cell surface expression of MHC I. However, CatG along with LF triggered an increase of MHC I (Figure 2B). Collectively, these findings identify LF as an enhancer of CatG-induced upregulation of MHC I.

### CatG increases MHC I on sphere-cultured stem cell-enriched cell populations (SCs)

**Figure 3:**
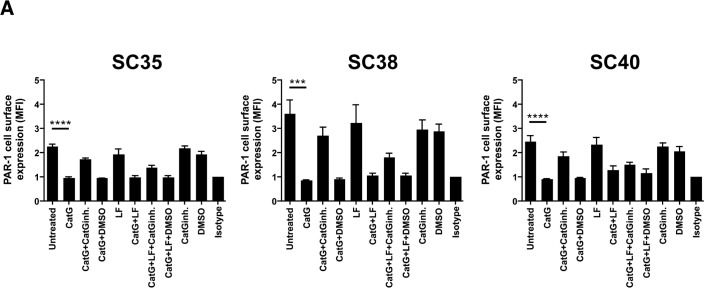

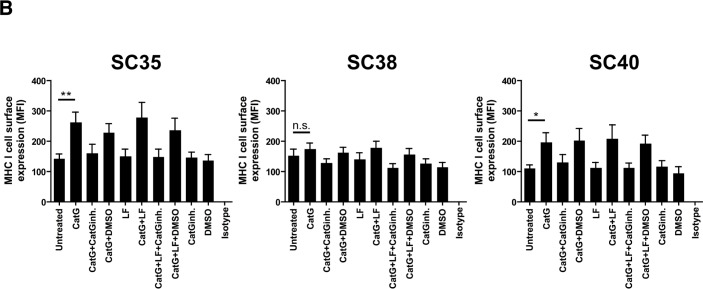

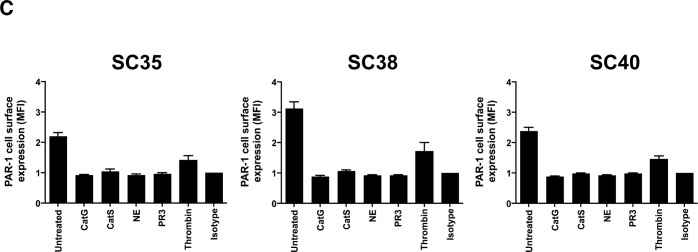

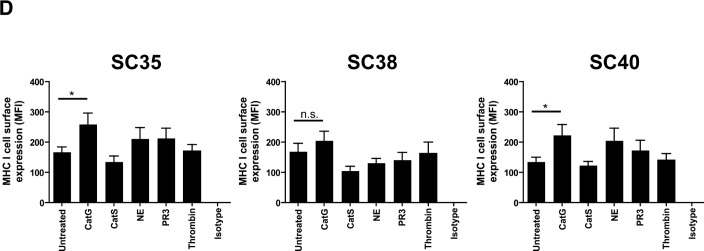
CatG induced cell surface expression of MHC I in spheres-cultured stem cell-enriched glioblastoma cell population (SC) **A.** SC35, SC38, and SC40 were incubated with CatG, LF, CatGinh., DMSO, CatG with LF, CatG with LF and CatG inhibitor (CatGinh.), CatG with LF and DMSO, or CatG with CatGinh. for 24h at 37°C. Cell surface expression of **A**. PAR1 or **B.** MHC I was determined by flow cytometry. All values were normalized to the isotype control. Eight independent experiments were performed (*n* = 8) and summarized in a bar diagram. SC35, SC38, and SC40 were treated with different proteases (CatG, CatS, NE, PAR3, and thrombin) for 24h at 37°C and levels of PAR1 **C.** or MHC I **D.** were analyzed by flow cytometry. Nine independent experiments were performed (*n* = 9) and values were normalized to the isotype control.

Next, we addressed the question whether CatG might upregulate MHC I in primary patient-derived glioblastoma stem cells. To this end, sphere-cultured stem cell-enriched cell populations (SCs) from three different glioblastoma patients (SC35, SC38, and SC40) were incubated with CatG and levels of PAR1 and MHC I were assessed by flow cytometry. While PAR1 was downregulated in all SCs tested (Figure 3A), MHC I was significantly upregulated in SC35 and SC40 and glioblastoma cell line (U87) but not in SC38 (Figure 3B and Supp. Data S4). Notably, SC35, SC38, and SC40 did not harbor PAR2, PAR3, and PAR4 at the cell surface (Supp. Data S5 to S7). Although CatS, NE, PR3, and thrombin downregulated PAR1 these proteases did not significantly alter expression of MHC I in SC35, SC38, and SC40 (Figure 3C and 3D), which might account for their different proteolytic cleavage sites. Taken together, CatG can induce MHC I in primary patient-derived glioblastoma stem cells.

### Immune cells force target cells to display their MHC I at the cell surface

**Figure 4:**
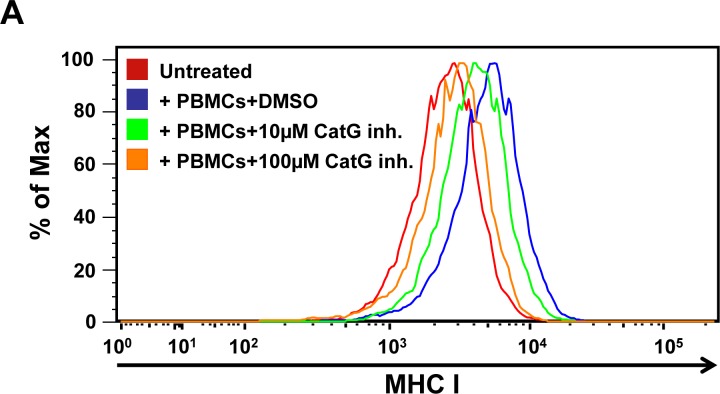

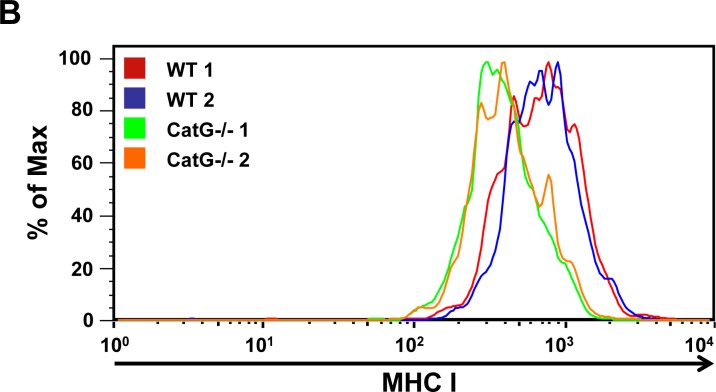
Functional assay for CatG-mediated enhancement of cell surface MHC I molecules **A.** PBMCs were co-cultured with THP-1. To trace THP-1 cells in co-culture with PBMCs, THP-1 were permanently labeled with CellTrace Violet cytoskeleton dye. Cell surface levels of MHC I were analyzed. Three independent experiments with similar results were performed. **B.** Spleen DCs from wild-type (WT) and CatG^−/−^ mice were analyzed for their MHC I cell surface expression. Data from one of two independent experiments is shown (*P* < 0.05; median MFI WT 634, CatG^−^/− 433, *n* = 4/genotype).

To ascertain the physiological relevance of the CatG-induced increase of MHC cell surface expression, we co-cultured freshly isolated PBMCs with THP-1. PBMCs harbor T cells, NK cells, activated pro-inflammatory monocytes, and B cells, altogether known to bind CatG to their cell surfaces [10, 11, 24]. The presence of cell surface CatG on PBMCs was confirmed by a CatG specific antibody and flow cytometry (Supp. Data S8). CellTrace Violet-labeled THP-1 cells showed abundant levels of MHC I, when co-cultured with PBMCs, and this effect was abrogated by using the CatG inhibitor (Figure 4A). Moreover, phorbol 12-myristate 13-acetate (PMA), which is known to induce CatG secretion in granulocytes [25], provokes an increase of CatG and MHC I on PBMCs (Supp. Data S9) as well as in activated platelets (Supp. Data S10), and CatG enhances the secretion of IFN-g (Supp. Data S11).

Both exogenous and cell surface bound CatG were responsible for upregulation of MHC I and might facilitate communication between immune cells. In order to test this hypothesis under physiological conditions, we analyzed the cell surface expression of MHC I in CatG deficient professional antigen presenting cells such as dendritic cells (DCs). Splenocytes were isolated from either CatG-deficient (CatG−/−) or control wild-type (WT) mice and cell surface expression of MHC I was analyzed in CD11c^+^-DCs. We detected reduced levels of cell surface MHC I in CD11c^+^-DCs isolated from CatG−/− mice compared to their WT counterparts (Figure 4B). This reduction of MHC I can be restored by incubating DCs from CatG deficient mice with CatG (Supp. Data S12). Thus, the absence of CatG *in vivo* alters levels of MHC I in DCs.

## DISCUSSION

The data provided, demonstrate that exogenous CatG upregulates MHC I on the cell surface of different cell types and the concert action of LF and CatG revealed further upregulation of MHC class I antigens on PBMCs and the B cell line BSM but not on glioblastoma stem cells. Furthermore, PBMCs, which exhibit CatG on the cell surface, induce cell surface expression of MHC I molecules on THP-1, which explains the finding why DCs from CatG-deficient mice harbor less MHC I at the cell surface then DCs from wild type mice.

Our findings suggest the following scenario regarding a novel role for CatG in controlling cell surface expression of MHC I. CatG is a potent chymotrypsin-like serine protease produced and released by neutrophils and contributes to killing microbes as well as processing host proteins, including extracellular matrix and cell surface receptors [26, 27]. Free CatG activity is rapidly inhibited by secreted plasma serpins such as alpha1-antitrypsin, anti-chymotrypsin, and secretory leukocyte protease inhibitor (SLPI) [28, 29]. Similarly, intracellular granule leakage of CatG within the neutrophil cytoplasm is rapidly inhibited by Serpinb1 to prevent neutrophil death [30]. In contrast, cell-surface bound CatG cannot be reached by these large inhibitors due to steric hindrance [24]. T cells, NK cells, and B cells can bind CatG to their cell surface [10, 11], where CatG retains its activity. These immune cells communicate with antigen presenting cells (APCs) and generate an excessive proteolytic microenvironment between CatG and cell-cell contact side, which provokes an increased expression of MHC molecules on APCs in a mechanism that might synergize with inhibition of protease-activated receptor 1 (PAR1) signaling. As a result, cytotoxic T cells might be activated as demonstrated in Supp. data S11. Alternatively, APCs may be in contact with free CatG at sites of inflammation where proteases are in excess of their anti-protease shield. While the PARs activating protease (thrombin) and tcY-NH_2_ did not alter levels of MHC I, CatG and the PAR1 antagonist FR enhanced cell surface expression of MHC I by using PBMCs as target cells. Thus, CatG-mediated induction of MHC expression might depend on an alternative PAR1 signaling pathway since PAR1 probably slows down MHC I expression. Glioblastoma stem cells partly down modulate their MHC I cell surface expression and infiltrate to the surrounding brain tissue thereby escape immune recognition [7]. We found that CatG also upregulates MHC I on glioblastoma stem cells (SCs) which is certainly important for CTL recognition. However, glioblastoma cells secrete protease inhibitors [31] which can diminish the proteolytic activity of CatG and might support the immune evasion of glioblastoma stem cells.

In addition, the presented data provide further evidence for cooperation of LF and CatG in innate immunity. According to the recent report, we demonstrate the augmentation of the enzymatic activity of CatG by LF [17]. Here, the results precisely indicate an enhancing effect of LF to CatG in upregulation of MHC I at the cell surface of PBMCs. Although LF or CatG alone do not affect MHC class I expression to the B cell line BSM but when they act synergistically MHC I expression was enhanced.

In conclusion, we demonstrate that CatG effectively upregulates cell surface MHC I suggesting that CatG is essential for post-transcriptional regulation of MHC I molecules and for intercellular communication between immune cells and target cells.

## MATERIALS AND METHODS

### Animals

Mice deficient in CatG (CatG^−/−^, *Ctsg^tm1Ley^*) in C57BL6 background (Christine Pham, Washington University, St Louis, MO, USA) were co-housed and age- and sex-matched C57BL/6J wild-type (WT) mice were used as controls. Animal studies were approved by the Cantonal Veterinary Office of Bern and conducted in accordance with the Swiss federal legislation on animal welfare. Mouse splenocytes were incubated with labeled antibodies against CD11c-PE (clone N418), CD45-PerCP (clone 30-F11), MHC-I-APC (clone AF6-88.5) (BioLegend, San Diego, CA, USA) and analyzed by flow cytometry on a FACSCalibur (BD Biosciences, Minneapolis, MN, USA). Relative mean fluorescence intensity (MFI) of MHC-I on dendritic cells (CD45^+^CD11c^+^) was determined using FlowJo (Tree Star, Inc., Ashland, OR, USA) and MFI of MHC-I from 4 mice/genotype was analyzed by Mann Whitney U test using Prism 6.0c (GraphPad. San Diego, CA, USA).

### Assessment of MHC I surface expression on THP-1 cells

THP-1 cell line [32] was cultured in RPMI 1640 medium supplemented with 10% fetal bovine serum (FBS) and antibiotics. Cells were washed with PBS pH 7.4 and incubated with either the protease-activated receptor 1 (PAR1) antagonist, FR171113 (10 μM, Tocris, Bristol, UK) [22], or PAR4 antagonist, (*trans*-Cinnamoyl)-YPGKF-NH_2_ (tcY-NH_2_, 10 μM, Tocris) [23] with or without CatG for 6 h at 37°C. DMSO served as a vehicle control. Subsequently, cells were washed in PBS and stained with anti-HLA-ABC-APC (clone W6/32; eBioscience, San Diego, California, USA) diluted in blocking buffer (1% FBS in PBS) for 30 min at 4°C. After two wash steps, cells were fixed in PBS containing 2% paraformaldehyde (PFA) and subsequently measured by a BD Canto II cytometer (Franklin Lakes, NJ, USA). Data were acquired and analyzed using FlowJo software (Tree Star, Inc., Ashland, OR, USA).

### CatG-induced MHC I cell surface expression in PBMCs

B cell line (BSM) or cryopreserved PBMCs from healthy donors (young, 18-25 years and elderly, 59-70 years) were used for the 6h assay. PBMCs (2×10^6^ cells/ml) were incubated with CatG (8 μg/ml; from human neutrophils, Enzo, Life Sciences or BioCentrum Ltd., Krakow, Poland or from human sputum, Sigma-Aldrich, München, Germany), heat inactivated CatG (98°C for 15 min), recombinant CatS (0.1 μg/ml, Enzo, Life Sciences), or human recombinant LF from rice (250 μg/ml, MyBioSource, San Diego, CA, USA). To determine CatG specificity, CatG was preincubated with CatG inhibitor I (100 μM, Calbiochem, Schwalbach, Germany), Suc-Val-Pro-Phe^P^ (OPh)_2_ (Suc-VPF, 100 μM; Jozef Oleksyszyn, Faculty of Chemistry, Wroclaw University of Technology, Wroclaw, Poland), or the cysteine protease inhibitor E64 (100 μM, Enzo Life Sciences). In a separate experiment, PBMCs were incubated with PAR1 antagonist, FR171113 (10 mM, Tocris) for 6 h at 37°C. DMSO served as a vehicle control. Cells were washed in PBS containing 1% FBS and stained with anti-HLA-ABC-APC (clone W6/32; eBioscience) or the isotype control (mouse IgG2a APC, eBioscience) for 30 min at 4°C and cells were measured by FACSCalibur (BD Biosciences). Separately, cells were extracellular stained with anti-human PAR1-PE (25 ug/ml, R&D Systems, Abingdon, UK) or isotype control (mouse IgG2B, PE conjugated antibody, R&D Systems, Abingdon, UK) and analyzed by flow cytometry. Data were acquired and analyzed using FlowJo software (Tree Star). Bar diagram and statistical analysis were done by GraphPad Prism 4 (GraphPad Software, Inc., San Diego, CA, USA). Use of PBMCs for *in vitro* studies is in accordance with the local ethics committee (approved proposal # 327/14).

### Induction of MHC I expression in THP-1 by PBMCs

THP-1 cell line was cultured in RPMI 1640 medium supplemented with 10% FBS and antibiotics. Cells were washed with PBS pH 7.4. THP-1 cells were permanently labeled by using the CellTrace Violet Cell proliferation kit (Invitrogen, Carlsbad, CA, USA) according to the manufacturer's protocol in order to allow identification of THP-1 cells after co-culture. 100,000 labeled THP-1 and 20,000 freshly isolated PBMCs were co-cultured in serum-free RPMI medium in the presence or absence of the CatG-inhibitor (10μM or l00μM, Calbiochem) for 24 h. Then cells were stained with anti-HLA-ABC-APC or anti-HLA-DR-PE and subjected to flow cytometry as described above. Cells positive for CellTrace Violet were gated and analyzed. Studies involving human material were reviewed and approved by the University of Ulm Institutional Review Board, and individuals and/or their legal guardians provided written informed consent prior to donating blood. Use of PBMCs for *in vitro* studies is in accordance with the local ethics committee (approved proposal # 327/14).

### Glioblastoma cell line

Human glioblastoma cell line U87-MG (U87, American Type Culture Collection, Manassas, VA, USA) were cultured in DMEM supplemented with 10% FBS and 1% penicillin (120 mg/ml)/streptomycin (120 mg/ml) (Life Technologies).

### Sphere-cultured stem cell-enriched glioblastoma cell populations (SCs)

Astrocytoma grade IV tissue from three different patients (No. 35, 38, or 40) was minced, washed in PBS, and incubated with TrypLE Express (Gibco, Life Technologies). Cells were filtered and cultured in DMEM/F-12 medium (Gibco, Life Technologies) containing L-glutamine, 0.01% (v/v) epidermal growth factor (EGF, Biomol GmbH, Hamburg, Germany), 0.04% (v/v) fibroblast growth factor (FGF, Miltenyi Biotec, Bergisch Gladbach, Germany), 1% (v/v) B27 (Gibco, Life Technologies), 2% Fungizone (Gibco, Life Technologies), 1% penicillin (120 mg/ml)/streptomycin (120 mg/ml) (Life Technologies) [33]. These cells are herein determined as a sphere-cultured stem cell-enriched glioblastoma cell populations (SCs). Stem cell and differentiation markers were expressed accordingly [34]. Use of SCs is in accordance with the local ethics committee at Ulm University (# 162/10).

### CatG-induced MHC I expression in SCs

Cells were incubated with CatG, CatG with CatG inhibitor I (50 μM), CatG with lactoferrin (LF, 250 mg/ml ~3.27 mM; from rice, MyBioSource, San Diego, CA, USA), bovine thrombin (8 μg/ml, Sigma-Aldrich), NE (8 μg/ml, Elastin Products Company, Owensville, MO, USA), PR3 (8 μg/ml, Elastin Products Company, Owensville, MO, USA), CatG and LF with CatG inhibitor I, or CatG and LF with DMSO for 24 h at 37°C. Cell surface MHC I or PAR1 (anti-human PAR1, PE conjugated, monoclonal mouse IgG2B, R&D Systems, Abingdon, UK) was determined by flow cytometry as described above.

### Statistical analysis

Data were depicted as median ± standard error of the median (S.E.M.) and statistical analysis was performed using the unpaired, two-tailed Student's *t*-test (Prism 4, GraphPad Software, La Jolla, CA, USA).

## SUPPLEMENTARY MATERIALS FIGURES



## ABBREVIATIONS

APCs: antigen-presenting cells
BSM: B cell line (B-lymphoblastoid cells)
Cat: cathepsin
DC: dendritic cell
ER: endoplasmatic reticulum
HLA: human leukocyte antigen
MFI: mean fluorescence intensity
MHC: major histocompatibility complex
PARs: protease-activated receptors
PBMCs: peripheral blood mononuclear cells
PMA: phorbol 12-myristate 13-acetate
SucVPF: Suc-Val-Pro-Phe^P^ (OPh)_2_
THP-1: human acute monocytic leukemia cell line
