# Supplementary material for: Exogenous cathepsin G upregulates cell surface MHC class I molecules on immune and glioblastoma cells
Source: Oncotarget. 2016 Oct 28;7(46):74602–11. doi: 10.18632/oncotarget.12980 (PMC5342690; doi:10.18632/oncotarget.12980)
Supplement: Supplementary file 1 [file oncotarget-07-74602-s001.pdf]

Exogenous cathepsin G upregulates cell surface MHC class I molecules on immune and glioblastoma cells

Supplementary Material

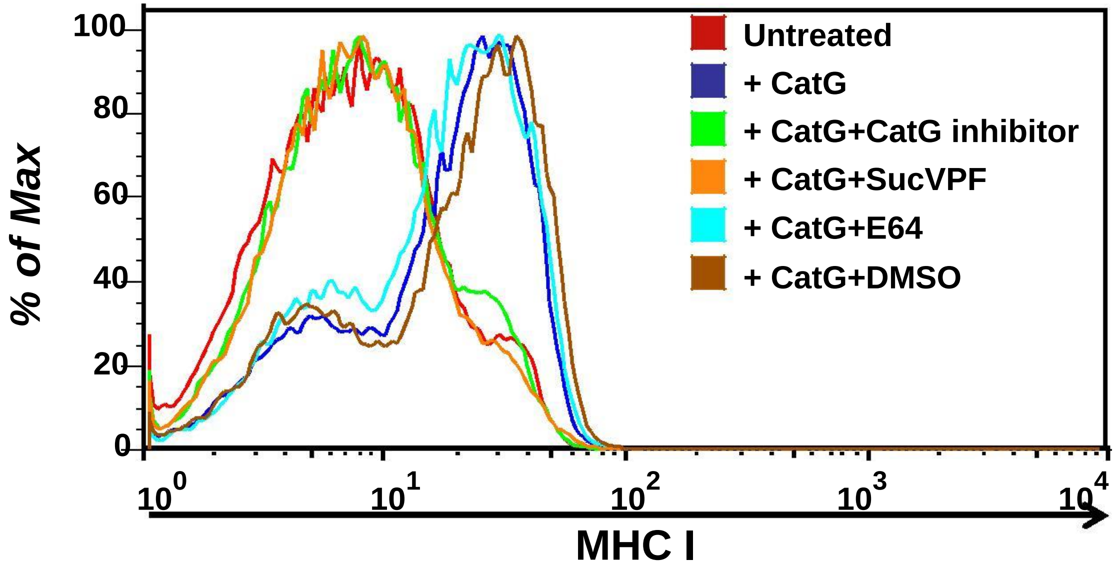

Supplementary Figure 1:

THP-1 were incubated (6 h) with purified CatG with or without CatG inhibitor, Suc-VPF (irreversible CatG inhibitor), cysteine protease inhibitor, or DMSO. Samples were stained with anti -HLA-ABC-APC. Cell surface levels of MHC I was analyzed by flow cytometry. Results are representative of three independent experiments.

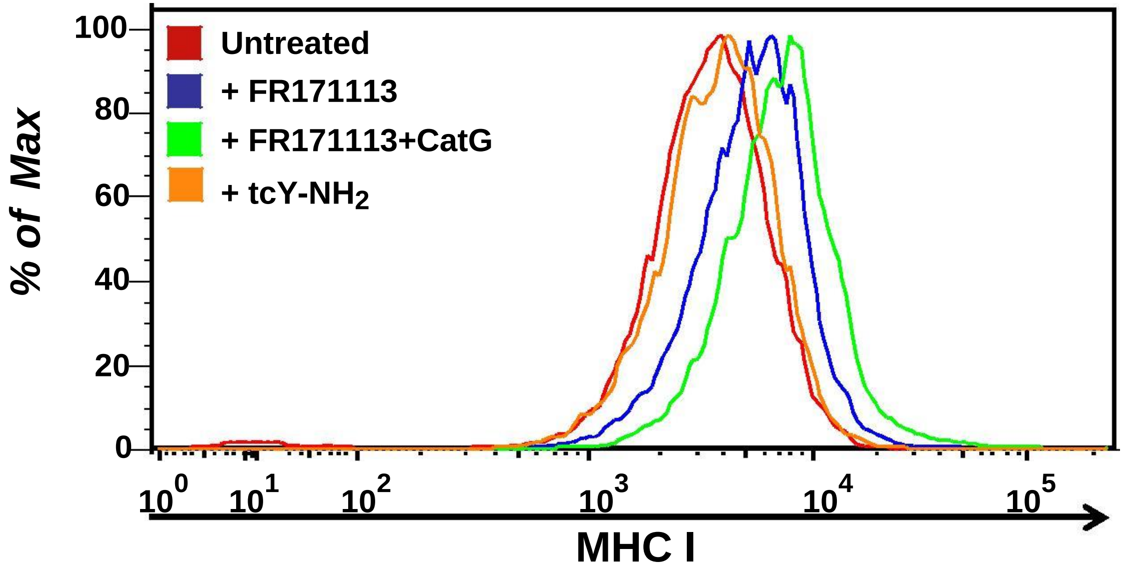

Supplementary Figure 2:

Protease-activated receptor 1 (PAR1) antagonist upregulates MHC I.

THP-1 were incubated with either the protease-activated receptor 1 (PAR1) antagonist (FR171113) with or without CatG and cell surface expression was determined. The PAR4 antagonist (*trans*-Cinnamoyl)-YPGKF-NH_2 (tcY-NH_2) was used as a control. Two independent experiments.

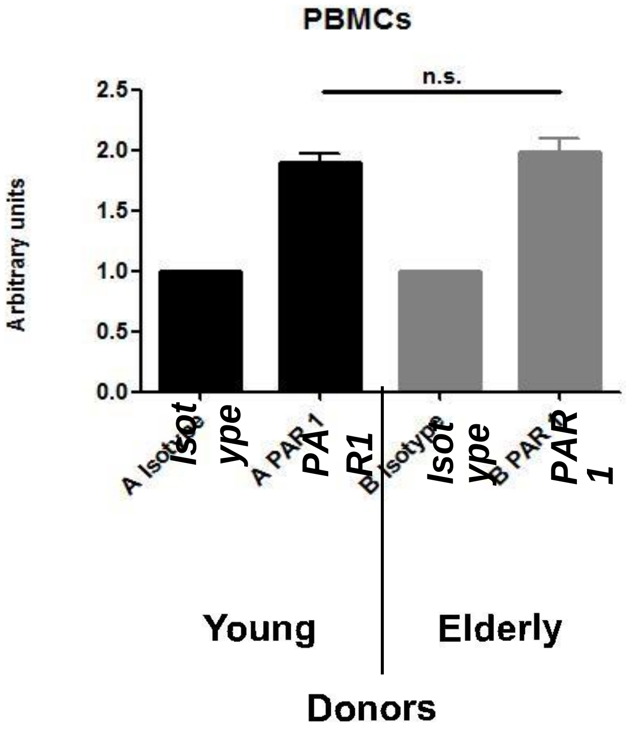

Supplementary Figure 3:

Protease-activated receptor 1 (PAR1) detection on PBMCs.

PBMCs were incubated with anti-human PAR1PE conjugated (R&D Systems, Abingdon, UK) and analyzed by flow cytometry. Young donors, n=10 ; elderly donors, n=10.

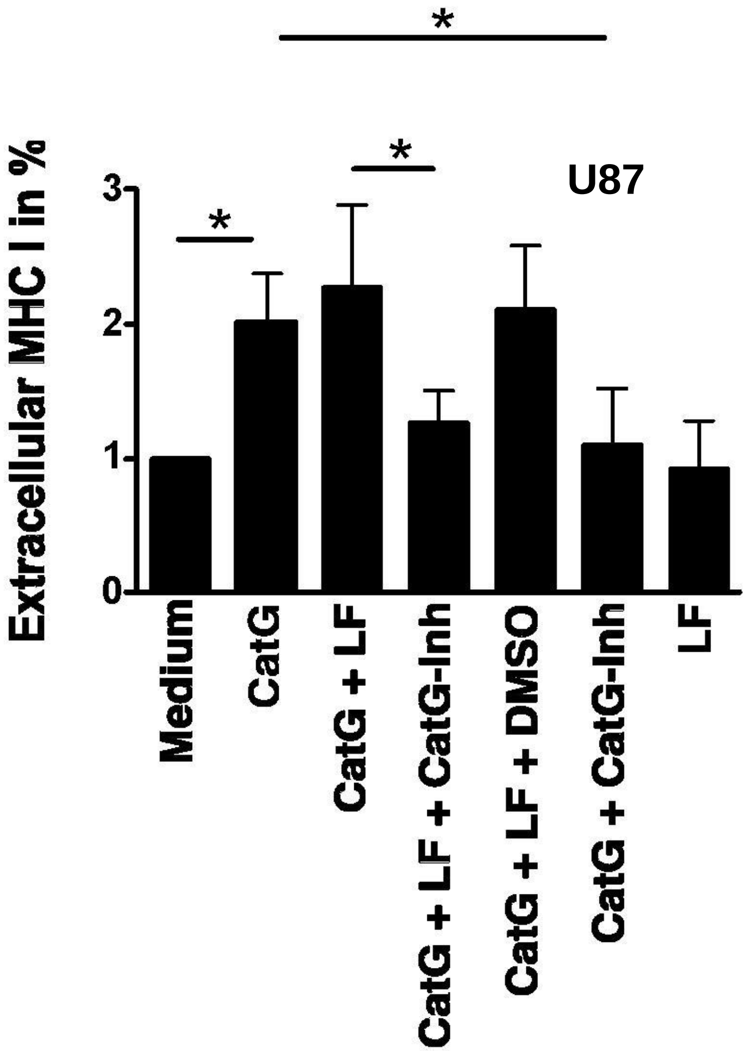

Supplementary Figure 4:

Human glioblastoma cell line, U87, were cultured with or without CatG, CatG with CatG inhibitor I (100 μM), CatG with lactoferrin (LF, 250 μg/ml ~3.27 μM; from rice, MyBioSource, San Diego, CA, USA), CatG and LF with CatG inhibitor I, or CatG and LF with DMSO for 6 h at 37° C. The expression of cell surface MHC I was analyzed by flow cytometry. Four independent experiments is summarized in the bar diagram.

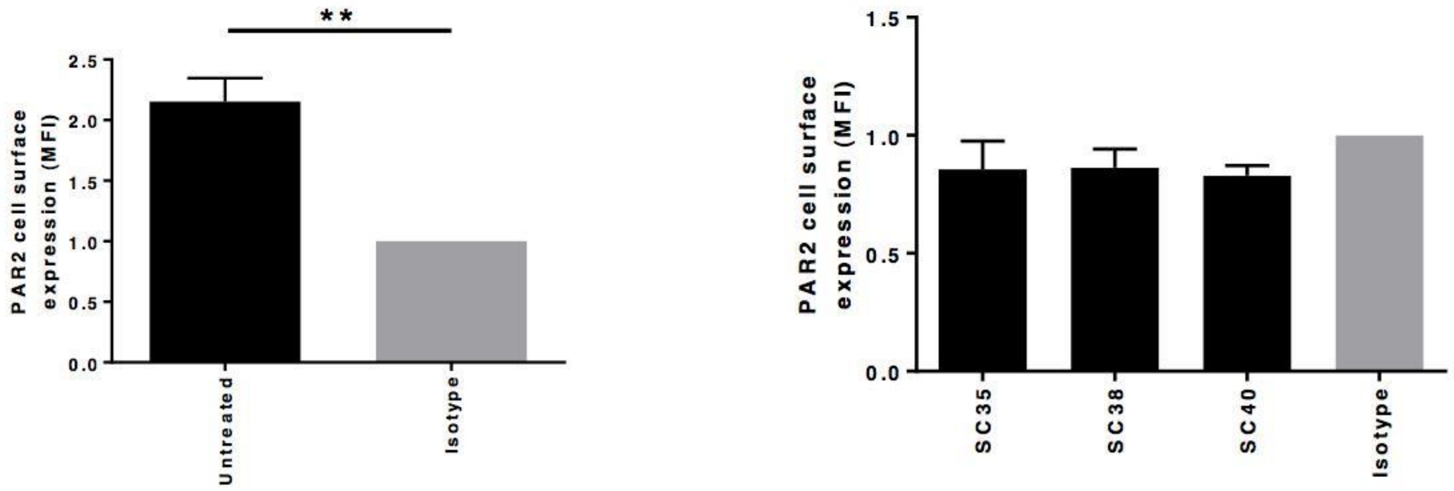

Supplementary Figure 5:

Protease-activated receptor 2 (PAR2) detection on PBMCs and SCs.

PBMCs, SC35, SC38, and SC40 were incubated with anti-human PAR2 (IgG2A) conjugated with APC (R&D Systems, Abingdon, UK) and analyzed by flow cytometry. PBMCs, n=4 and SCs, n=3.

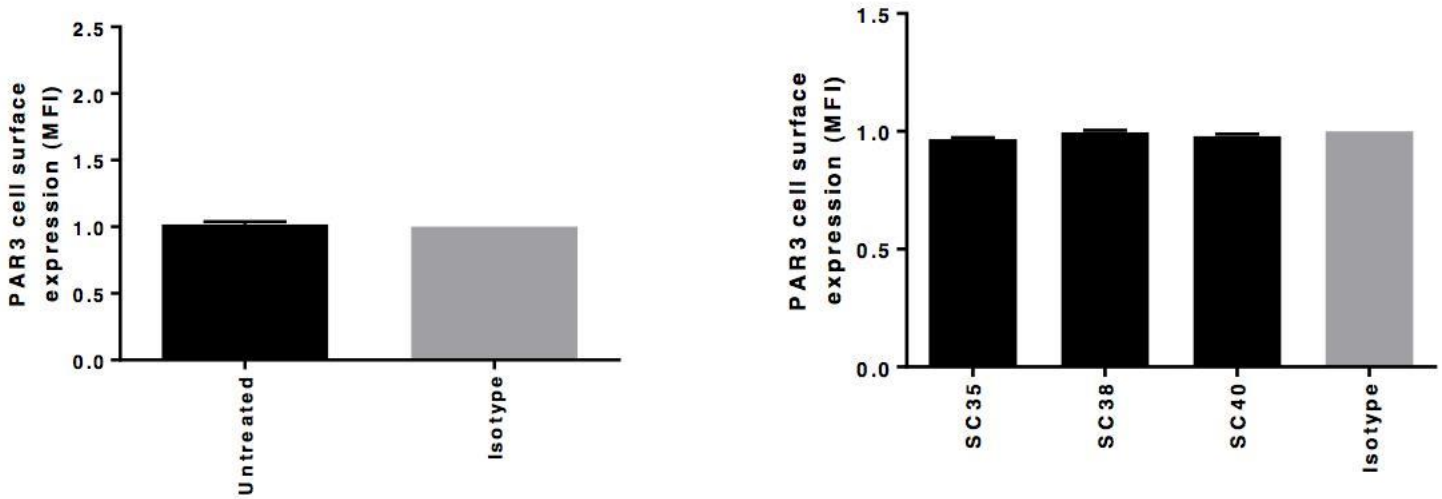

Supplementary Figure 6:

Protease-activated receptor 3 (PAR3) detection on PBMCs and SCs.

PBMCs, SC35, SC38, and SC40 were incubated with anti-human PAR3 (IgG2B)-FITC (Santa Cruz Biotechnology, Dallas, Texas, USA) and analyzed by flow cytometry. PBMCs, n=4 and SCs, n=3.

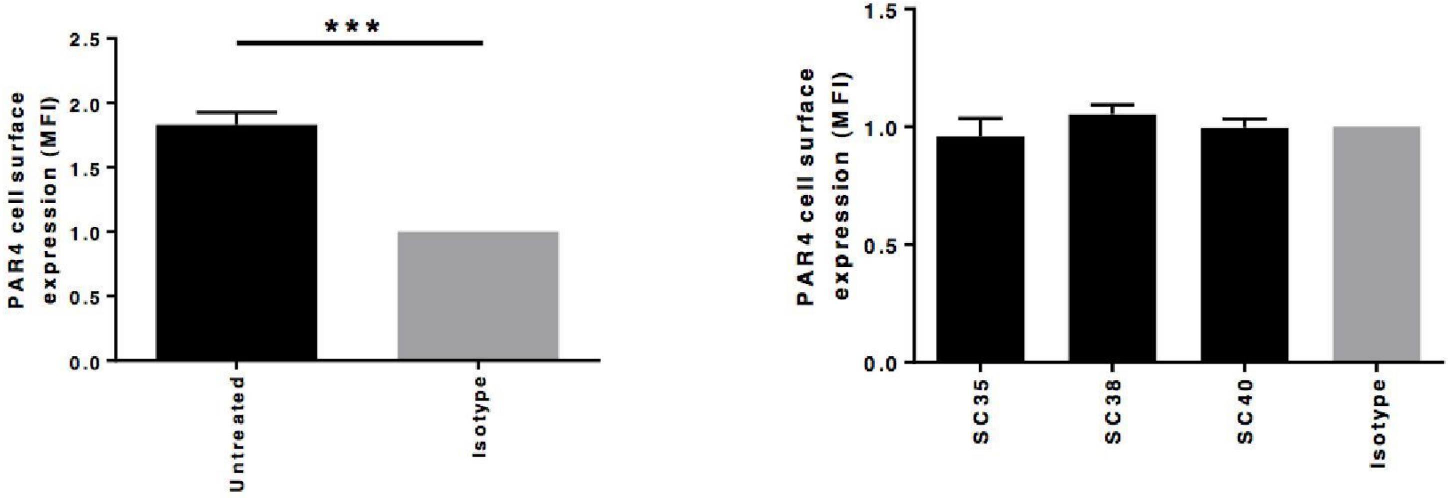

Supplementary Figure 7:

Protease-activated receptor 4 (PAR4) detection on PBMCs and SCs.

PBMCs, SC35, SC38, and SC40 were incubated with anti-human PAR4-FITC (Alomone labs, Jerusalem, Israel) and analyzed by flow cytometry. PBMCs, n=3 and SCs, n=3.

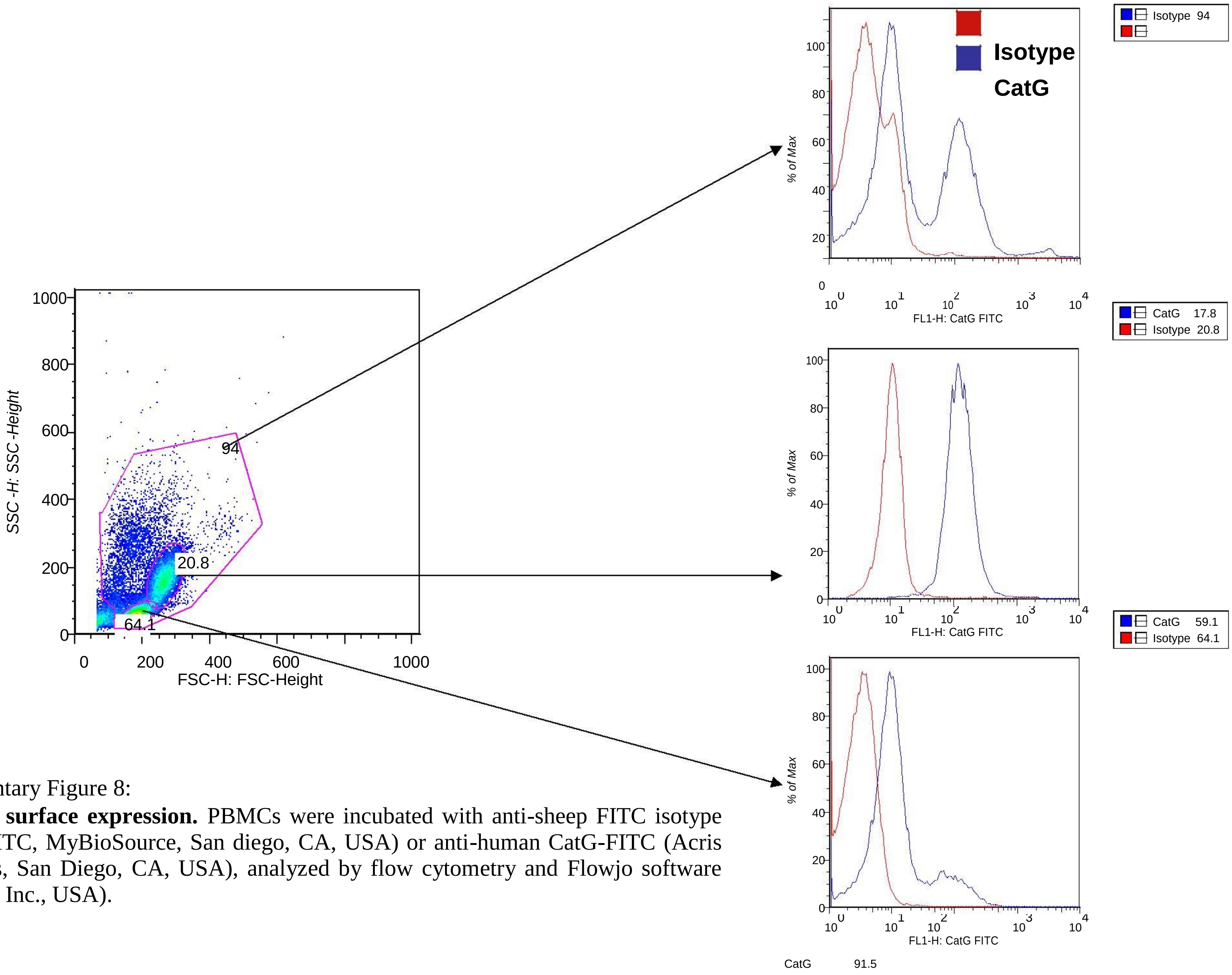

Supplementary Figure 8:

CatG cell surface expression. PBMCs were incubated with anti-sheep FITC isotype control (FITC, MyBioSource, San diego, CA, USA) or anti-human CatG-FITC (Acris Antibodies, San Diego, CA, USA), analyzed by flow cytometry and Flowjo software (Tree Star, Inc., USA).

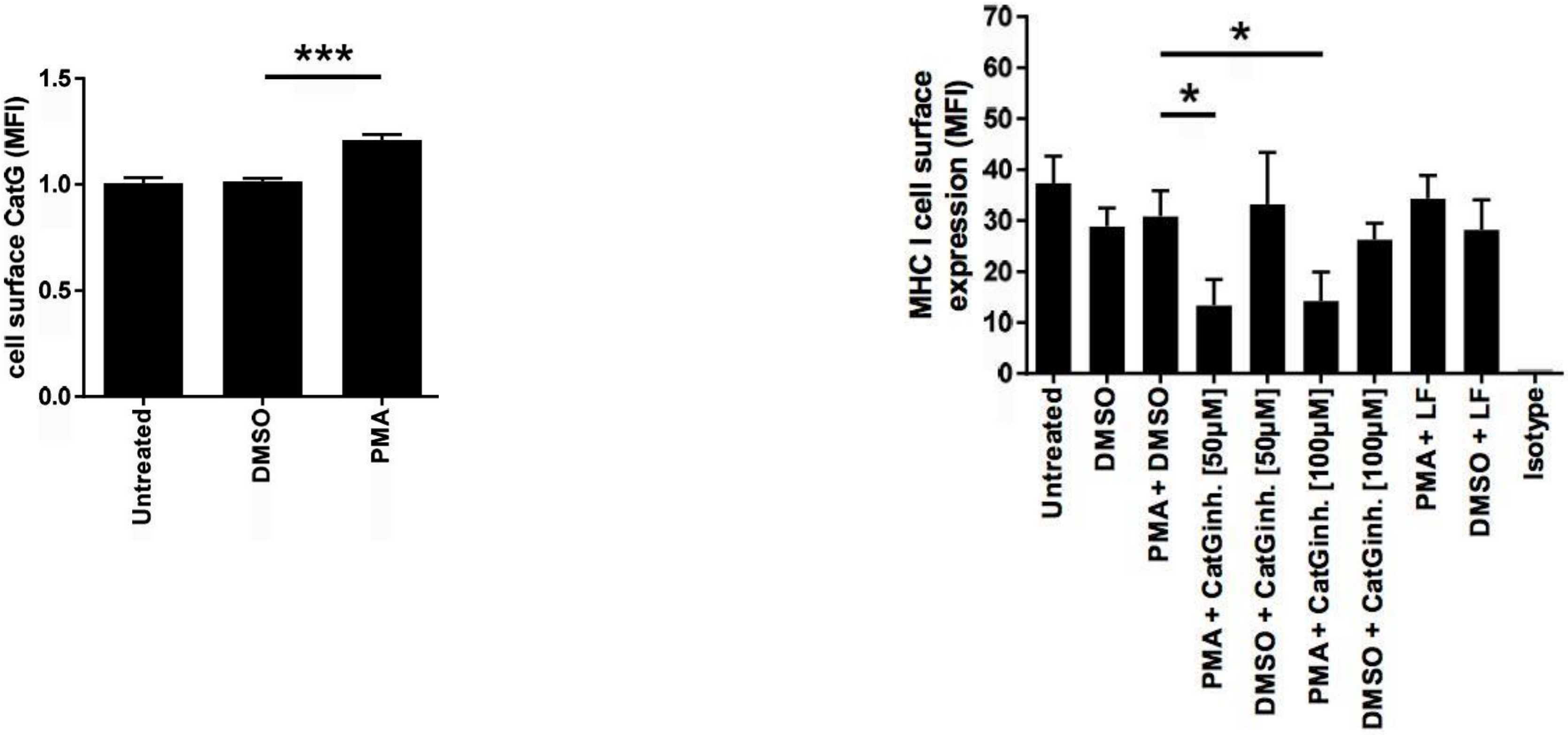

Supplementary Figure 9:
PMA provokes an increase of CatG and MHC I on PBMCs.

It is known that PMA induces the secretion of CatG in granulocytes. PBMCs and granulocytes were separated from peripheral blood and then co-cultured with granulocytes. Cells were treated with the CatG inhibitor or vehicle control (DMSO) for 6 h at 37C. Cell surface levels of CatG after stimulation with PMA (left panel) or levels of MHC I (right panel) was analyzed by flow cytometry. n=7.

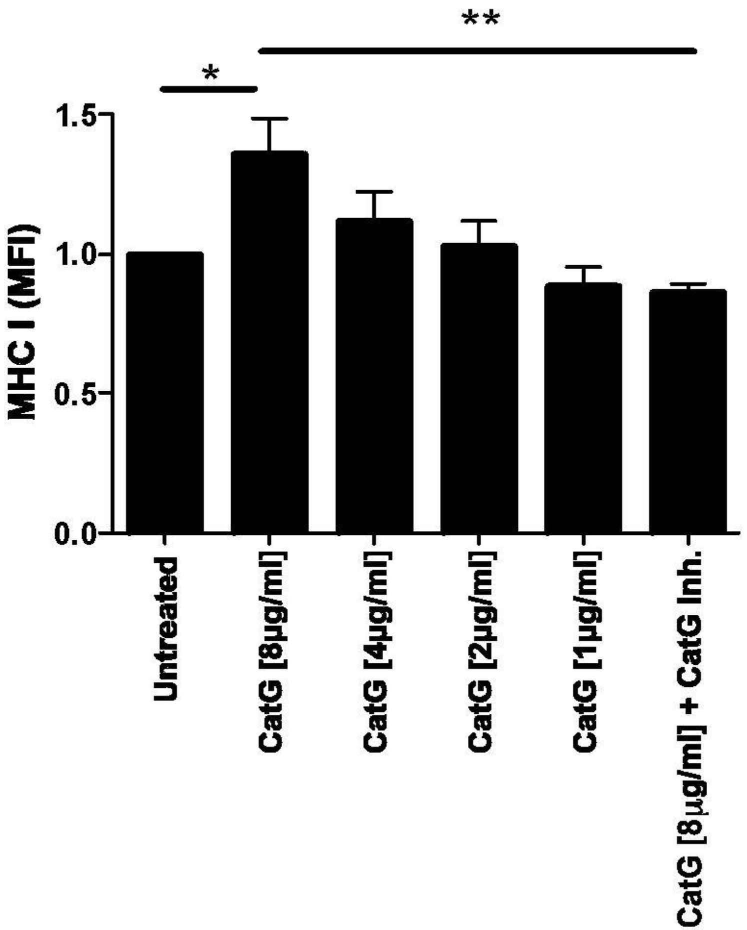

Supplementary Figure 10:

Platelet activation assay.

Primary human platelets (buffy coat) were incubated with CatG, which is known for its platelet activation capacity, for 30 min and platelet activation was determined by CD62P (platelet activation marker). Levels of cell surface MHC I were increased by CatG. In total n=5 donors. CatG Inh. = CatG inhibitor.

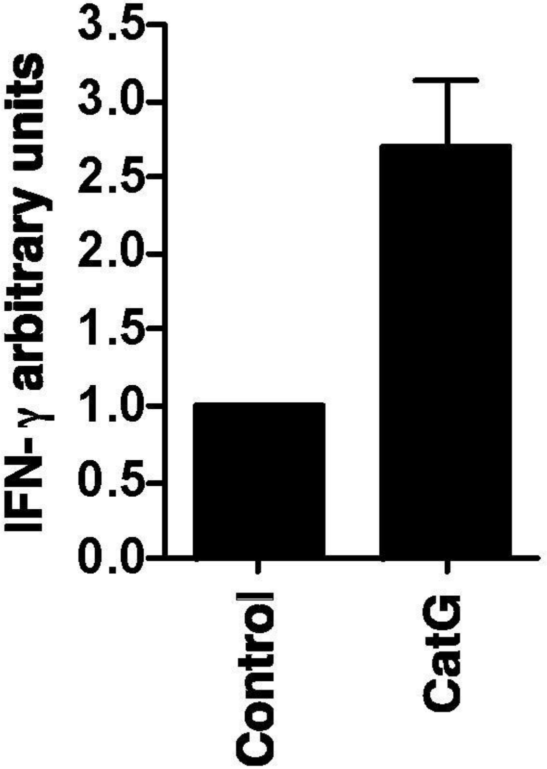

Supplementary Figure 11:

CatG increases the secretion of IFN-γ .

PBMCs were incubated with CatG for five days at 37 ° C. Supernatant were collected to perform IFN-γ-specific ELISA. Data were normalized. In total n=7 donors.

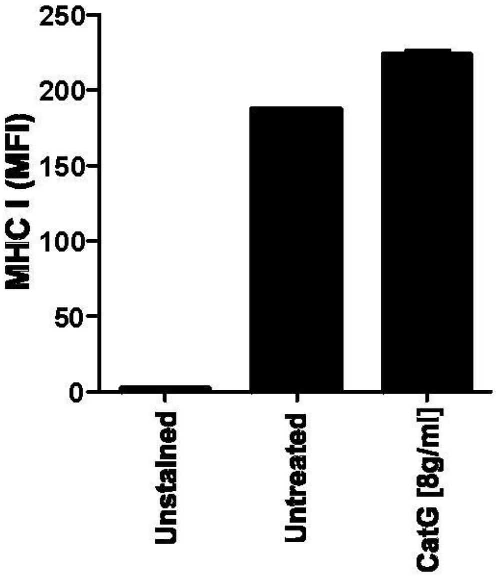

Supplementary Figure 12:

CatG increases levels of MHC I on the cell surface of DCs from CatG deficient mice .

Spleen cells were incubated with human CatG (8µg/ml) for 5h at 37° C. Anti-MHC I-PE, -CD45-PerCP, and -CD11c-APC was purchased from eBiosciences (Frankfurt, Germany). Levels of MHC I was determined by flow cytometry. One experiment out of three is shown.
